# A data-driven typology of asthma medication adherence using cluster analysis

**DOI:** 10.1038/s41598-020-72060-0

**Published:** 2020-09-14

**Authors:** Holly Tibble, Amy Chan, Edwin A. Mitchell, Elsie Horne, Dimitrios Doudesis, Rob Horne, Mehrdad A. Mizani, Aziz Sheikh, Athanasios Tsanas

**Affiliations:** 1 Usher Institute, Edinburgh Medical School, College of Medicine and Veterinary Medicine, University of Edinburgh, Doorway 1, Old Medical School, Teviot Place, Edinburgh, EH8 9AG UK; 2 Asthma UK Centre for Applied Research, Usher Institute, Centre for Medical Informatics, University of Edinburgh, Edinburgh, UK; 3 School of Pharmacy, Faculty of Medical and Health Sciences, University of Auckland, Auckland, New Zealand; 4 Centre for Behavioural Medicine, Department for Practice and Policy, UCL School of Pharmacy, University College London, London, UK; 5 Department of Paediatrics: Child and Youth Health, Faculty of Medicine and Health Sciences, University of Auckland, Auckland, New Zealand; 6 BHF Centre for Cardiovascular Sciences, University of Edinburgh, Edinburgh, UK; 7 Health Data Research UK, London, UK

**Keywords:** Data mining, Risk factors, Epidemiology, Asthma

## Abstract

Asthma preventer medication non-adherence is strongly associated with poor asthma control. One-dimensional measures of adherence may ignore clinically important patterns of medication-taking behavior. We sought to construct a data-driven multi-dimensional typology of medication non-adherence in children with asthma. We analyzed data from an intervention study of electronic inhaler monitoring devices, comprising 211 patients yielding 35,161 person-days of data. Five adherence measures were extracted: the percentage of doses taken, the percentage of days on which zero doses were taken, the percentage of days on which both doses were taken, the number of treatment intermissions per 100 study days, and the duration of treatment intermissions per 100 study days. We applied principal component analysis on the measures and subsequently applied k-means to determine cluster membership. Decision trees identified the measure that could predict cluster assignment with the highest accuracy, increasing interpretability and increasing clinical utility. We demonstrate the use of adherence measures towards a three-group categorization of medication non-adherence, which succinctly describes the diversity of patient medication taking patterns in asthma. The percentage of prescribed doses taken during the study contributed to the prediction of cluster assignment most accurately (84% in out-of-sample data).

## Introduction

Asthma is a long-term condition that can be effectively managed in the majority of individuals through regular use of inhaled corticosteroids (ICS)^1–3^. *Medication non-adherence*, that is not taking a medication as prescribed, is however very common and is a major factor associated with poor asthma control, leading to increased likelihood of asthma attacks^4–8^. Sub-optimal adherence is a costly problem due to medication waste and unnecessary additional healthcare utilization to treat uncontrolled symptoms that may have arisen from non-adherence^9–14^.

Numerous studies have used targeted interventions to improve medication adherence in patients with asthma, with the aim of improving clinical outcomes (such as reducing the risk of asthma symptoms or attacks). While some studies have shown positive changes in adherence behavior, they are usually short in duration, making their true clinical and cost-effectiveness hard to assess^15–25^.

Electronic monitoring devices (EMDs) enable the real-time tracking of inhaler use, by means of a small electronic chip fitted onto an inhaler, which records the date and time of each dose taken. EMDs are commonly used in research studies aimed at measuring adherence to inhaler asthma therapy^26^, as they can track the exact time and frequency of inhaler actuations. EMDs can thus measure medication use more accurately than either self-report or indirect adherence measurement methods such as using prescribing or dispensing records, which makes their data a valuable resource for investigating patterns of adherence behaviors.

There are many ways of measuring medication non-adherence and the correlation between these measures within the same data is heavily context dependant^27,28^. There is currently no consensus on what should be considered the gold standard^27–30^. Furthermore, despite the multitude of medication non-adherence measures^31^, many intervention studies describe medication adherence using only a single average measure over time (such as reporting the percentage of days on which the prescribed doses of ICS were taken^32–36^), without supporting rationale for their choice.

Previous studies of adherence using EMDs in asthma have most commonly used the proportion of prescribed doses that were taken^34,37–40^, or the percentage of days on which adherence was deemed acceptable by the authors, according to timing of doses^38,41^.

Adherence is often reported categorically by dichotomizing individuals into “good” and “poor” adherers, according to a binary threshold, commonly 80%^40,42–50^. This threshold, based on a hypothesised level of adherence required to maintain therapeutic effect, which may be considered arbitrary, as any such level would be condition and drug-specific^51^ and rely on consistency in the space between doses over time^52–54^. Very few studies have attempted a more detailed and meaningful dissection, such as categorising based on trends over time^55,56^, or by assessing multiple components of adherence^7,57,58^.

This study aimed to investigate the underlying patterns of medication non-adherence in children with asthma, and to identify multidimensional behavior subgroups.

## Methods

### Data sources

We undertook a secondary analysis of data from a previously published randomized controlled trial (RCT)^37^. Chan et al.^37^ investigated the effect of an EMD inhaler with an audio-visual reminder function (timed alarm) on ICS adherence. The study enrolled children who presented to the emergency department for acute asthma symptoms and were diagnosed with asthma by the emergency department medical team. The diagnosis process would usually include a clinical diagnosis based on the presentation and medical assessment, supported by pulmonary function tests.

There were 220 children enrolled, aged 6–15 years, of whom 110 were randomized to the control group, and 110 to the intervention group. Both groups received the same EMD; however, in the intervention group, the EMD had the audio-visual function enabled, whilst the control group had this function disabled. The device was provided to study participants at the baseline appointment, and data from each device was collected at each of the three follow-up appointments (approximately week 8, week 16, and week 24).

The original study inclusion criteria were: children between 6 and 15 years, with physician-diagnosed asthma, presenting to the emergency department with an asthma attack, and on twice-daily ICS treatment (with or without additional asthma treatments). Diagnosis of a chronic lung disease other than asthma, or congenital heart disease, led to exclusion from participating in the study, as well as living outside of the Auckland, New Zealand catchment area.

### Measures of adherence

In chronic disease management, it is common for patients to take *intermissions* of treatment (periods of consecutively missed doses), followed by *re-initiations* (returning to treatment following an intermission). As such, it is possible to categorise adherence measures into the subdomains of *implementation* (dose-taking within a day^31^) and *persistence* (the incidence and duration of treatment intermissions lasting at least five days, as discussed by Andrade et al.^59^).

Twice daily asthma medications are typically taken in the morning and evening, to ensure consistent bioavailability throughout a 24-h period. As such, we refer herein to a ‘dose’ as one or more actuations (puffs) of the inhaler during a 12-h period (midnight to midday, and midday to midnight). Each individual in this study, by virtue of the inclusion criteria, was on a twice daily regimen, and thus had two potential doses in a day (before and after midday). Herein we define five measures of adherence used in this analysis, calculated at the person-level, with denominators excluding periods in which the electronic inhaler device was malfunctioning. Their algorithmic definitions are as follows:

The percentage of doses that were taken:

A $$ \frac{{\text{Doses taken}}}{{2 \times {\text{Days with device}}}} \times 100 $$

The percentage of days on which zero doses were taken:

B $$ \frac{{\text{Days on which zero doses were actuated}}}{{\text{Days with device}}} \times 100 $$

The percentage of days on which both doses were taken:

C $$ \frac{{\text{Days on which doses were actuated in both the AM and PM}}}{{\text{Days with device}}} \times 100 $$

The number of treatment intermissions, per 100 days of follow-up:

D $$ \frac{{\text{Number of treatment intermissions}}}{{\text{Days with device}}} \times 100 $$

The duration of time spent on treatment intermissions, per 100 days of follow-up:

E $$ \frac{{\text{Total duration of time spent on treatment intermission}}}{{\text{Days with device}}} \times 100 $$

### Data analysis

Using principal component analysis (PCA), we extracted the *latent variables* of the adherence measures*.* Latent variables (also known as *principal components*) are linear functions of the original measures, which identify the underlying structure of the measures and avoid redundant information^60^. We identified the minimum number of principal components required to explain at least 95% of the variance between the measures.

Principal components can be influenced by varying magnitudes of scale (e.g. range 0–1 versus range 0–1,000) in the input features. As a benchmark, we used only *zero-centering* (subtracting the mean from each value) to pre-process the adherence measures prior to applying PCA. We also compared the computed principal components generated from the adherence measures when the measures underwent different pre-processing before applying PCA: (1) after scaling each variable to *unit variance* (subtracting the mean from each value, and dividing by the variance), and (2) using *min–max scaling* (each variable was scaled to lie between 0 and 1).

We used k-means clustering with 25 random initiations, searching through for the optimal number *k* of clusters (we searched for *k* = 1, 2, …, 15). We visually assessed the within groups sum of squares using a *scree plot*^61^, to determine the optimal number of clusters. For each method of scaling/centering applied to the data in the principal component computation, we performed clustering on bootstrapped samples of the principal components, repeating the process 1,000 times to assess the stability of the resulting clusters.

The optimal scaling method was selected as the one that led to the most stable clusters, formally assessed using measures derived from the Jaccard similarity coefficient.

The Jaccard coefficient of cluster solution similarity^62^ is the mean Jaccard similarity between (1) the clusters constructed from the full sample, and (2) the corresponding cluster constructed from bootstrapped samples. The cluster in the bootstrap sample corresponding to the original cluster is determined as the one with the highest Jaccard similarity to the original cluster.

In this process, a bootstrap sample cluster is considered *dissolved* when the Jaccard similarity between the full data cluster and the associated cluster from a bootstrap sample is less than 0.5^63^. As such, we can also measure the cluster dissolution rate, the proportion of bootstrap samples for which the cluster dissolved.

Finally, we trained standard classification and regression trees (CARTs)^60^, using each of the measures described above to estimate the adherence groups, using the computed cluster labels as ground truth. The trees were constructed using the Breiman et al. algorithm^64^ with the following parameters:

- At least 20 patients must be at a node for a split to be considered,
- There must be at least 6 patients at any terminal node,
- The maximum tree height is 2 nodes,

tenfold cross-validation was conducted to estimate the optimal *complexity parameter* (threshold improvement in model fit required for a split to be retained) for use in the tree pruning in order to minimize the cross-validated error rate. The tree is then pruned according to this estimated complexity parameter.

The tree was built on 70% of the data (randomly sampled). A partition of 15% was used to generate a confusion matrix for each measure; mapping the combinations of cluster labels and tree labels, to quantify the quality of the estimates produced by the decision tree. The best measure was selected by highest accuracy was identified, as follows:

$$ {\text{Accuracy}} = \frac{{\text{Number of correctly classified patients}}}{{\text{Number of patients}}} $$

We then used the remaining 15% of the data (the test set) to report the final accuracy.

All analyses were conducted in R version 3.5.1, and scripts for data cleaning, setup and analysis will be made available on GitHub, a platform which host open-source projects (https://github.com/hollytibble/Electronic-Monitoring-Device-Adherence-Typology).

### Ethics

Ethics approval was granted by the New Zealand Northern Y Regional Ethics Committee (NTY/08/12/116) and District Health Boards. Verbal and written informed consent was obtained at data collection from the parent or guardian of all participants. Analysis and reporting in this study was performed in accordance with the relevant guidelines and regulations.

## Results

### Data cleaning and missing data

A total of 220 children were enrolled into the study. There were nine participants (seven from the control group and two from the intervention group) who did not complete the study. Reasons for drop-out were loss to follow-up (n = 5), missing data from one or more follow-up appointment (n = 2), non-compliance with protocol (n = 1) and study withdrawal (n = 1). This left a total of 211 participants with data from all study follow-up periods for inclusion in this analysis, yielding 36,902 person-days of data.

There were 1,741 person-days of missing data, out of a total 36,902 person-days (5% missing). This was due to battery failure, indicated in the device log, rather than simply individuals missing doses. 55 (26%) children in the study sample had at least one day with missing data, with a median 22 days missing (interquartile range (IQR): 10–48, range 2–122) from a median 185 days of follow-up (IQR: 176–190, range: 155–210). These periods of missing data were removed, resulting in 35,161 person-days for analysis.

The peak hours for actuations were 7–10 AM (96% of actuations between midnight and midday) and 7–10 AM (93% of actuations between midday and midnight).

### Study population

The study population consisted of 108 boys and 103 girls, aged between 6 and 15 (median 8) years. The most common ethnicity was White European origin (38%; eTable 1).

### Adherence

**Figure 1:**
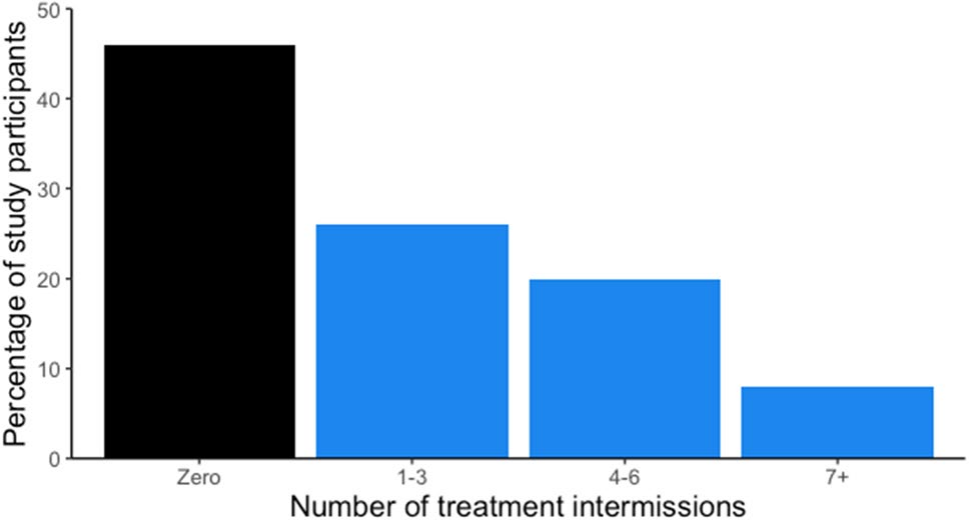
Histogram of the number of treatment intermissions (continuous abstinence of 5 days or longer) per person during study period.

In the control group, 36% of prescribed doses were taken, compared to 80% in the intervention group. 54% of the overall cohort (n = 114) had at least one treatment intermission (Fig. 1), with the first starting a median of 42 days after the baseline visit (IQR 14–95 days). Individual’s first treatment intermissions had a median duration of eight days (IQR 6–14 days).

The difference between the intervention arms was marked, with 27% (n = 29) of the intervention group participants having at least one treatment intermission, compared to 83% (n = 85) in the control group.

### Exploratory cluster analysis

The correlations between the adherence measures used herein have previously been reported^65^. For each scaling method, the scree plot consistently found three as the most appropriate number of clusters. Computing the principal components from the unit-variance scaled measures gave the most robust solution (eTable 2). Table 1 shows the variable loadings for the first four principal components.

**Table 1:** Principal components to identify the latent variable structure of (unit-scaled) medication non-adherence.

	PC1	PC2	PC3	PC4
(A) Percentage of doses taken	0.98	− 0.18	− 0.03	0.02
(B) Percentage of days on which zero doses were taken	− 0.99	− 0.04	0.10	− 0.09
(C) Percentage of days on which both doses were taken	0.93	− 0.36	0.04	− 0.05
(D) Number of treatment intermissions per 100 study days	− 0.94	− 0.28	0.19	0.07
(E) Duration of treatment intermissions per 100 study days	− 0.93	− 0.23	− 0.29	0.00
Percentage of variance explained	91%	6%	< 1%	< 1%

### Determining clinically meaningful subgroups

Statistics to summarize the distribution of the measures by cluster are presented in eTable 3. Cluster 1 had the poorest adherence—taking on average (between individuals median) 16% of their prescribed doses and approximately 4.0 treatment intermissions of five or more days in length for every 100 days of follow-up. In Cluster 3, only 7 individual (11%) took any treatment intermissions, and on average they took 91% of their doses. For each measure, Cluster 2 was somewhere between the first and third clusters.

**Figure 2:**
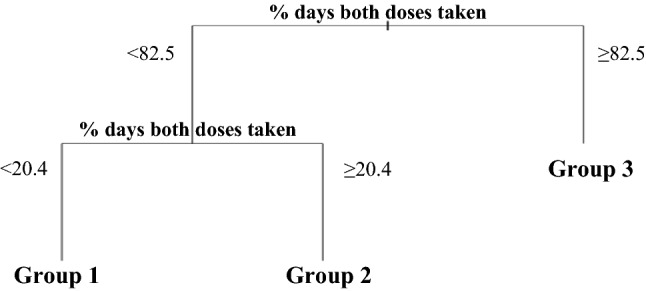
Decision tree for estimating exploratory adherence clusters.

**Figure 3:**
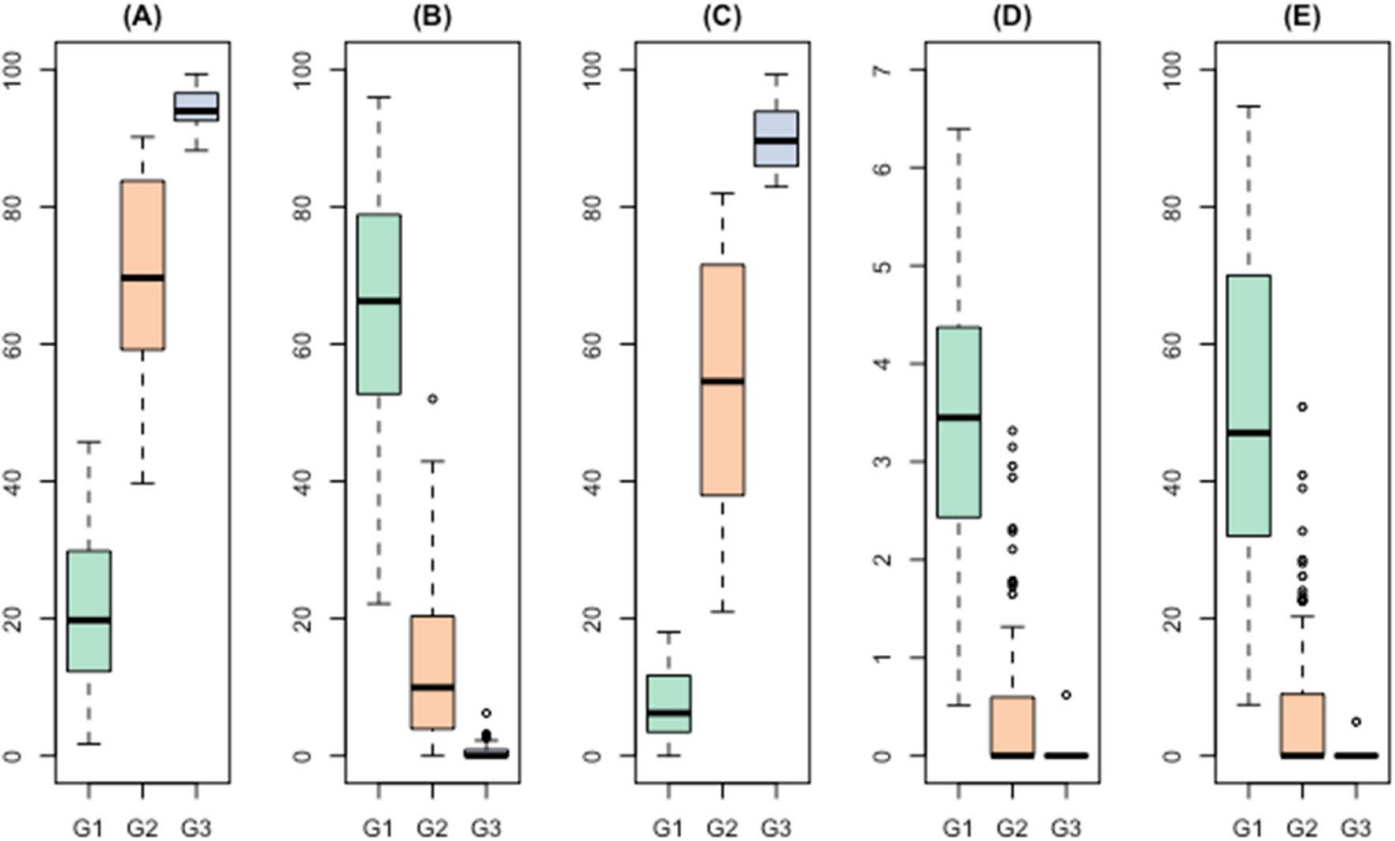
Boxplots of the five adherence measures by tree-derived groups. (**A**) Percentage of doses taken, (**B**) Percentage of days zero doses taken, (**C**) Percentage of days both doses taken, (**D**) Number of treatment intermissions per 100 study days, (**E**) Duration of treatment intermissions per 100 study days.

We constructed a decision tree for each adherence measure in isolation, using the cluster labels as the response variable, in a standard supervised learning set-up framework. We used a random 70% sample of the data as a training set and the remaining 30% as a testing set. The percentage of doses taken yielded the highest accuracy (i.e. the group using the decision tree matching the group computed using the exploratory clustering approach) at 88%. The resulting decision tree (Fig. 2) had an accuracy of 84% in the test set (Table 2). Figure 3A–E present the boxplots of the measures by group.

**Table 2:** Confusion matrix using the automatically determined cluster labels (used as 'ground truth') and CART estimates in the testing set.

n = 32	Exploratory clusters		
	C1	C2	C3
**CART groups**			
G1	**4**	2	0
G2	1	**18**	0
G3	0	0	**5**

## Discussion

In this study, we identified three exploratory clusters of adherence, which were found to be very stable across the 1,000 bootstrapped repetitions to assess cluster stability. These clusters, representing poor, moderate, and good medication adherence, were best approximated in a decision tree using the measure of the percentage of prescribed doses that were taken during the study (84% accuracy in data unseen to the training model).

Due to the limited follow-up time, we were required to aggregate adherence measures over the duration of the study. It is likely that with a larger sample and a longer follow-up we may have observed transitioning between states of behavior, perhaps triggered by clinical events such as an asthma attack. This study used data from 211 children, and hence findings should be interpreted tentatively: it is possible that having more samples would enable finer stratification and exploration of additional clusters.

Some individuals with asthma are instructed to take their medication ‘as needed’, as they are able to maintain adequate asthma control despite taking medicine inconsistently. We did not remove such children from the population, as their adherence patterns remain of interest. We were additionally unable to stratify the population accordingly due to the small sample size. In a larger study, it might have been that different groups were detected according to the strictness of their regimen, or that observed groups occurred in different frequencies.

The proportion of days on which both daily doses were taken was used as one of the measures of adherence. This measure was calculated assuming that one dose (of at least one actuation) was taken between midnight and midday, and one between midday and midnight. For the most part, this meant one dose in the morning, and one in the evening, as is usually recommended. However, if a dose was taken at 11.30 in the morning, followed by another at 12.30 in the afternoon, this would be recorded as having taken both the morning and evening doses, despite their being insufficient time between the doses for optimal distribution throughout the day. In this cohort, over 96% of morning and 93% of evening doses were taken within a 3-h window in each period (between 7:00–10:00 o’clock in the morning window, and between 19:00–22:00 in the afternoon window). As such, this is unlikely to have been a common concern in the context of this study.

Finally, this study was undertaken in a narrow cohort with narrowly defined inclusion criteria i.e. children on a twice daily regimen for clinician-diagnosed asthma, with a recent history of emergency care presentation for an asthma attack. The generalizability of these findings to all children diagnosed with asthma, (or adults diagnosed with asthma) would therefore need to be further studied.

### Future work

There is a need to study adherence patterns in adults with asthma, as well as in children (and adults) in primary care setting rather than only using a cohort of those with asthma related emergency department presentations. As technology advances and the cost of manufacturing EMDs decreases, we envisage an increase in their adoption in clinical care^66,67^, which may mean that future large scale studies can be conducted on routine practice datasets.

There are no studies which have attempted data driven categorisation, as far as we are aware. A recent study by Allemann et al. generated simulated pharmacy refill records according to six proposed adherence subtypes and attempted to detect them using data driven trajectory-based models^68^. Trajectory-based models offer little scope for the integration of domain-specific knowledge. As the authors note, this means that identifying clusters which are clinically interpretable and meaningful according to their temporal patterns is challenging. While electronic monitoring devices are considered the gold standard for adherence measurement in asthma, adapting our methods to refill data may result in the identification of some of the clusters described by the authors. Furthermore, the application of such trajectory-based models in a (larger) electronic monitoring device dataset could potentially uncover some previously unidentified subgroups.

## Conclusions

Medication taking behavior in children with asthma is highly heterogeneous, and there is no consistent pattern of a patient with poor adherence. Through analysis of multiple adherence measures, we identified subgroups of asthma patients according to data-driven thresholds. Our findings suggest that the simple measurement of percentage of doses taken is sufficient in this population, and that combinations measures of non-adherence are unlikely to be of further clinical value than any of these measures in isolation.

## Supplementary information

Supplementary information 1

Supplementary information 2

## Abbreviations

CART: Classification and regression tree
EMD: Electronic monitoring device
ICS: Inhaled corticosteroids
IQR: Interquartile range
PC: Principal component
PCA: Principal component analysis
RCT: Randomized controlled trial
US: United States
