# Supplementary material for: A data-driven typology of asthma medication adherence using cluster analysis
Source: Sci Rep. 2020 Sep 14;10:14999. doi: 10.1038/s41598-020-72060-0 (PMC7490405; doi:10.1038/s41598-020-72060-0)
Supplement: Supplementary file 1 — Supplementary information 1 [file 41598_2020_72060_MOESM1_ESM.docx]

## A Data-Driven Typology of Asthma Medication Adherence using Cluster Analysis

**Authors:** Holly Tibble ^1,2,*^, Amy Chan^3,4^, Edwin A Mitchell^5^, Elsie Horne ^1,2^, Dimitrios Doudesis ^1,6^, Rob Horne ^2,4^, Mehrdad A Mizani ^1,2^, Aziz Sheikh ^1,2,7^, Athanasios Tsanas ^1,2^

rm(list=ls()) # remove all variables from workspace

# Package installations

library(readxl)

library(plyr)

library(dplyr)

library(foreign)

library(data.table)

library(reshape2)

library(tidyr)

library(ggplot2)

library(corrplot)

library(stringr)

library(fpc)

library(rpart)

library(lubridate)

library(openxlsx)

library(varhandle)

library(lme4)

library(cluster)

library(devtools)

library(ggbiplot)

library(RColorBrewer)

library(scales)

library(clue)

library(factoextra)

library(yardstick)

library(MASS)

library(randomForest)

library(wesanderson)

palette(alpha(brewer.pal(9,'Set1'), 0.5))

set.seed(12345)

###########################################################################

# Initiation

###########################################################################

### It could be either their first medication, or first period of missingness - if that came first

initiation<- data %>%

filter(Event %in% c("Medication","Data Missing")) %>%

arrange(StudyID,studyday,dose_time) %>%

group_by(StudyID) %>%

slice(1) %>%

mutate(first_med = ifelse(Event=="Data Missing",NA,studyday)) %>%

dplyr::rename(initiation_event = Event) %>%

dplyr::select(c("StudyID","first_med","initiation_event"))

###########################################################################

# Shell Setup

###########################################################################

### Get rid of all missing data records

data<-data[which(data$Event!="Data Missing"),]

# Work out how many days of non-missing data each person has (in total, and by month)

data<-data %>%

mutate(month = lubridate::month(first_day + days(studyday - 1))) %>%

group_by(StudyID,month) %>%

mutate(non_missing_days_month = max(row_number())/2) %>%

group_by(StudyID) %>%

mutate(non_missing_days = max(row_number())/2) %>%

ungroup()

# Create a shell to affix adherence measures to

Adherence <- data %>%

dplyr::select(StudyID,group,retention,non_missing_days) %>%

group_by(StudyID) %>%

slice(1)

Adherence<-left_join(Adherence, initiation)

rm(initiation)

###########################################################################

# Implementation

###########################################################################

### Day-level (looking at dose times split by morning and evening)

# daily rate of taking morning, evening, both or neither doses ***across cohort***

summary_implementation <- data %>%

dplyr::select(c("StudyID","studyday","dose_time","Freq","retention","non_missing_days")) %>%

gather(variable, value, c(Freq)) %>%

unite(temp, dose_time, variable) %>%

spread(temp,value) %>%

# drop the first day and the last day

filter(studyday!=1 & studyday!=retention) %>%

# add neither, both, Am or PM indicator

mutate(day_bin = ifelse(AM_Freq==0 & PM_Freq==0,"neither",

ifelse(AM_Freq>=1 & PM_Freq==0, "AM only",

ifelse(AM_Freq==0 & PM_Freq>=1,"PM only",

"both"))))

daily_implementation<-summary_implementation

### Day-level - want to know how behaviour changes across the whole population by time since initiation

summary_implementation<-summary_implementation %>%

group_by(studyday) %>%

dplyr::mutate(day_count=n()) %>%

group_by(studyday,day_bin) %>%

dplyr::mutate(category_day_count=n()) %>%

arrange(studyday,day_bin) %>%

slice(1) %>%

dplyr::mutate(category_day_pct = 100*category_day_count/day_count)

summary_implementation$day_bin<-as.factor(summary_implementation$day_bin)

summary_implementation$day_bin<-factor(summary_implementation$day_bin,

levels=c("neither","AM only","PM only","both"))

summary_implementation<-dplyr::select(summary_implementation,-c("StudyID","AM_Freq","PM_Freq","non_missing_days"))

### Person-level

daily_implementation<- daily_implementation %>%

mutate(day_freq = AM_Freq + PM_Freq) %>%

group_by(StudyID) %>%

#mutate(count_days_zero = sum(day_bin=="neither")) %>%

mutate(perc_doses = (sum(AM_Freq>0)+sum(PM_Freq>0))*100/(2*non_missing_days)) %>% # number of doses with SOME taken, over number of dose-times

mutate(perc_days_zero = sum(day_bin=="neither")*100/non_missing_days) %>%

mutate(perc_both = sum(day_bin=="both")*100/non_missing_days) %>%

#mutate(index = ifelse(day_bin!="neither",day_bin,NA)) %>%

#mutate(index_days = sum(!is.na(index))) %>%

#mutate(perc_both_non_zero = sum(day_bin=="both",na.rm=T)*100/index_days) %>%

slice(1) %>%

dplyr::select(-c("studyday","AM_Freq","PM_Freq","day_freq","day_bin"))

Adherence<-left_join(Adherence,daily_implementation)

rm(daily_implementation)

###########################################################################

# Persistence

###########################################################################

persistence <- data %>%

dplyr::select(StudyID,studyday,dose_time,Freq,Event,retention, non_missing_days) %>%

group_by(StudyID) %>%

# fifth_day_zero is true when the last 10 consecutive doses have been missed (5 days)

#(fifth_day_zero = 10<=((Freq==0)*sequence(rle(as.character(Freq==0))$lengths))) %>%

# how many days (0.5 days = 1 dose) in a row have they not taken any medicine

mutate(holiday_dur = (Freq==0)*sequence(rle(as.character(Freq==0))$lengths)/2) %>%

mutate(holiday_start_day = ifelse(holiday_dur>0 & dose_time=="AM",studyday-floor(holiday_dur),

ifelse(holiday_dur>0 & dose_time=="PM",studyday-floor(holiday_dur-0.5),

NA))) %>%

#mutate(holiday_censored = ifelse(row_number()==max(row_number()) & Event=="Medication Missed",1,NA)) %>%

filter(holiday_dur>=5) %>%

mutate(first_holiday_start = as.numeric(min(holiday_start_day))) %>%

mutate(first_holiday_dur = as.numeric(max(holiday_dur*(holiday_start_day==first_holiday_start)))) %>%

mutate(number_of_holidays = length(unique(holiday_start_day))) %>%

mutate(number_of_holidays_over_nmdays = length(unique(holiday_start_day))*100/non_missing_days) %>%

mutate(max_holiday_dur_over_nmdays = max(holiday_dur)*100/non_missing_days) %>%

group_by(StudyID,holiday_start_day) %>%

slice(n()) %>%

group_by(StudyID) %>%

mutate(total_holiday_dur = sum(holiday_dur)) %>%

mutate(total_holiday_dur_over_nmdays = sum(holiday_dur)*100/non_missing_days) %>%

dplyr::select(StudyID, number_of_holidays_over_nmdays, total_holiday_dur,

first_holiday_start,first_holiday_dur,number_of_holidays,

max_holiday_dur_over_nmdays,total_holiday_dur_over_nmdays) %>%

slice(n()) %>% ungroup

Adherence<-left_join(Adherence,persistence) %>%

replace_na(list(number_of_holidays=0,

total_holiday_dur=0,

number_of_holidays_over_nmdays=0,

total_holiday_dur_over_nmdays=0,

max_holiday_dur_over_nmdays=0))

rm(persistence)

Adherence$number_of_holidaysx<-ifelse(Adherence$number_of_holidays==0,"a.0",

ifelse(Adherence$number_of_holidays==1,"b.1",

ifelse(Adherence$number_of_holidays<=5,"c.2-5",

ifelse(Adherence$number_of_holidays<=10,'d.6-10',

"e.>10"))))

measure_list<-c("perc_doses","perc_days_zero","perc_both",

"total_holiday_dur_over_nmdays",

"number_of_holidays_over_nmdays")

###########################################################################

# Analysis Setup

###########################################################################

clusters <- Adherence[,measure_list]

clusters<-clusters %>% group_by()

rm(list=setdiff(ls(),c("clusters","measure_list")))

# Principal component analysis

clusters.pca <- prcomp(clusters, center = TRUE,scale. = TRUE)

summary(clusters.pca)

wss <- (nrow(clusters.pca$x[,1:2])-1)*sum(apply(clusters.pca$x[,1:2],2,var))

for (i in 2:10) wss[i] <- sum(kmeans(clusters.pca$x[,1:2],

centers=i)$withinss)

# Principal component analysis without scaling, for comparison

clusters.pca2 <- prcomp(clusters, center = TRUE,scale. = FALSE)

summary(clusters.pca2)

wss2 <- (nrow(clusters.pca2$x[,1:2])-1)*sum(apply(clusters.pca2$x[,1:2],2,var))

for (i in 2:10) wss2[i] <- sum(kmeans(clusters.pca2$x[,1:2],

centers=i)$withinss)

# MinMax scaling, for more comparison

norm <- function(x)

{

return((x- min(x)) /(max(x)-min(x)))

}

clusters_min_max<-as.data.frame(lapply(clusters, norm))

clusters.pca3 <- prcomp(clusters_min_max, center = FALSE, scale = FALSE)

summary(clusters.pca3)

wss3 <- (nrow(clusters.pca3$x[,1:2])-1)*sum(apply(clusters.pca3$x[,1:2],2,var))

for (i in 2:10) wss3[i] <- sum(kmeans(clusters.pca3$x[,1:2],

centers=i)$withinss)

###########################################################################

# Compare PCA variable scaling methods stability testing

###########################################################################

set.seed(12345)

cboot.hclust <- clusterboot(data.frame(clusters.pca$x[,1:2]),B=1000,clustermethod=hclustCBI,

method="ward.D", k=3, count = F, nstart=25)

cboot.hclust2 <- clusterboot(data.frame(clusters.pca2$x[,1:2]),B=1000,clustermethod=hclustCBI,

method="ward.D", k=3, count = F, nstart=25)

cboot.hclust3 <- clusterboot(data.frame(clusters.pca3$x[,1:2]),B=1000,clustermethod=hclustCBI,

method="ward.D", k=3, count = F, nstart=25)

# unit

round(cboot.hclust$bootmean,3)

cboot.hclust$bootbrd

# minmax

round(cboot.hclust3$bootmean,3)

cboot.hclust3$bootbrd

# none

round(cboot.hclust2$bootmean,3)

cboot.hclust2$bootbrd

set.seed(12345)

# Get PC loadings

round(get_pca_var(clusters.pca)$coord,2)

clusters_results<-clusters

clusters_results$groups<-ifelse(cboot.hclust$result$partition==1,"C2",

ifelse(cboot.hclust$result$partition==2,"C3",

"C1"))

tapply(clusters_results$perc_doses,clusters_results$groups,summary)

tapply(clusters_results$perc_days_zero,clusters_results$groups,summary)

tapply(clusters_results$perc_both,clusters_results$groups,summary)

tapply(clusters_results$number_of_holidays_over_nmdays,clusters_results$groups,summary)

tapply(clusters_results$total_holiday_dur_over_nmdays,clusters_results$groups,summary)

sum(clusters_results$groups=="C3" & clusters_results$number_of_holidays_over_nmdays>0)

sum(clusters_results$groups=="C3" & clusters_results$number_of_holidays_over_nmdays>0)*100/sum(clusters_results$groups=="C3")

###########################################################################

# CART

###########################################################################

# classifiers based on the clustering labels

fit <- rpart(data=clusters_results[which(clusters_results$set=="train"),],

groups ~ perc_both, method="class", maxdepth=2)

# prune and plot tree with clustering labels

pfit<- prune(fit, cp= fit$cptable[which.min(fit$cptable[,"xerror"]),"CP"])

plot(pfit, uniform=TRUE, main="Pruned Classification Tree")

text(pfit, use.n=TRUE, all=TRUE, cex=.8)

clusters_results$treegroup <- ifelse(clusters_results$perc_both<82.5,

ifelse(clusters_results$perc_both<20.4,

"G1",

"G2"),

"G3")

clusters_results$treegroup<-as.factor(clusters_results$treegroup)
