# Supplementary material for: A data-driven typology of asthma medication adherence using cluster analysis
Source: Sci Rep. 2020 Sep 14;10:14999. doi: 10.1038/s41598-020-72060-0 (PMC7490405; doi:10.1038/s41598-020-72060-0)
Supplement: Supplementary file 2 — Supplementary information 2 [file 41598_2020_72060_MOESM2_ESM.docx]

## A Data-Driven Typology of Asthma Medication Adherence using Cluster Analysis

**Authors:** Holly Tibble ^1,2,*^, Amy Chan^3,4^, Edwin A Mitchell^5^, Elsie Horne ^1,2^, Dimitrios Doudesis ^1,6^, Rob Horne ^2,4^, Mehrdad A Mizani ^1,2^, Aziz Sheikh ^1,2,7^, Athanasios Tsanas ^1,2^

## e-Supplement

**eTable 1: Demographics of the study population**

|  | Intervention (n=108) | | Control (n=103) |
| --- | --- | --- | --- |
|  | Number of participants (%) | | |
| Gender |  |  | |
| Female | 54 (50.0) | 49 (47.6) | |
| Male | 54 (50.0) | 54 (52.4) | |
| Age Group |  |  | |
| 6-8 | 53 (49.1) | 55 (53.4) | |
| 9-11 | 34 (31.5) | 28 (27.2) | |
| 12-15 | 21 (19.4) | 20 (19.4) | |
| Ethnicity |  |  | |
| Asian | 19 (17.6) | 19 (18.4) | |
| White European | 42 (38.9) | 38 (36.9) | |
| Maori | 6 (5.6) | 10 (9.7) | |
| Middle Eastern, Latin American or African (MELAA) | 2 (1.9) | 6 (5.8) | |
| Pacific Peoples | 23 (21.3) | 21 (20.4) | |
| Other | 16 (14.8) | 9 (8.7) | |
| New Zealand Index of Deprivation (centiles) |  |  | |
| Low (1-3) | 24 (22.2) | 24 (23.3) | |
| Medium (4-7) | 28 (25.9) | 25 (24.3) | |
| High (8-10) | 40 (37.0) | 38 (36.9) | |

**eTable 2: Cluster Stability Results by Variable Scaling Method**

|  | | Unit Variance Scaling | Min-Max Scaling | No  Scaling* |
| --- | --- | --- | --- | --- |
| Number of principal components | | 1 | 2 | 1 |
| % Variance explained | | 91.2% | 99.6% | 93.5% |
| Jaccard Similarity Index | Cluster 1 | 0.849 | 0.841 | 0.710 |
|  | Cluster 2 | 0.718 | 0.741 | 0.702 |
|  | Cluster 3 | 0.780 | 0.895 | 0.859 |
| Cluster Dissolutions | Cluster 1 | 0 | 0 | 173 |
|  | Cluster 2 | 75 | 143 | 107 |
|  | Cluster 3 | 0 | 0 | 5 |

* still zero-centered

**eTable 3: Variable summary distribution statistics by exploratory clusters**

| Adherence Measures | Cluster 1 (Poor) | Cluster 2 (Moderate) | Cluster 3 (Good) |
| --- | --- | --- | --- |
| (A) Percentage of doses taken | Median: 15.9  IQR: (9.2 – 22.5)  Range: (1.7 – 62.2) | Median: 59.9  IQR: (40.9 – 69.7)  Range: (18.6 – 84.1) | Median: 91.0  IQR: (87.2 – 94.3)  Range: (79.6 – 99.3) |
| (B) Percentage of days on which zero doses were taken | Median: 72.9  IQR: (62.3 – 81.9)  Range: (18.1 – 96.0) | Median: 19.7  IQR: (9.9 – 38.5)  Range: (0.0 – 66.4) | Median: 1.1  IQR: (0.0 – 2.7)  Range: (0.0 – 14.6) |
| (C) Percentage of days on which both doses were taken | Median: 4.8  IQR: (2.4 – 8.8)  Range: (0.0 – 45.7) | Median: 37.6  IQR: (21.0 – 54.8)  Range: (4.1 – 70.4) | Median: 84.3  IQR: (77.0 – 90.2)  Range: (67.5 – 99.3) |
| (D) Number of treatment intermissions per 100 study days | Median: 4.0  IQR: (3.2 – 4.6)  Range: (0.6 – 6.4) | Median: 0.6  IQR: (0.0 – 1.7)  Range: (0.0 – 3.3) | Median: 0.0  IQR: (0.0 – 0.0)  Range: (0.0 – 0.6) |
| (E) Duration of treatment intermissions per 100 study days | Median: 57.0  IQR: (45.8 – 71.7)  Range: (20.3 – 94.6) | Median: 6.5  IQR: (0.0 – 17.8)  Range: (0.0 – 45.2) | Median: 0.0  IQR: (0.0 – 0.0)  Range: (0.0 – 14.9) |
